# Genome-Wide Association Study on Adiponectin-Mediated Suppression of HDL-C Levels in Taiwanese Individuals Identifies Functional Haplotypes in *CDH13*

**DOI:** 10.3390/genes12101582

**Published:** 2021-10-07

**Authors:** Leay-Kiaw Er, Semon Wu, Tzuyu Cheng, Yu-Lin Ko, Ming-Sheng Teng

**Affiliations:** 1 Division of Endocrinology and Metabolism, Department of Internal Medicine, Taipei Tzu Chi Hospital, Buddhist Tzu Chi Medical Foundation, New Taipei city 23142, Taiwan; erleaykiaw@yahoo.com; 2 School of Medicine, Tzu Chi University, Hualien 97004, Taiwan; 3 Department of Life Science, Chinese Culture University, Taipei 11114, Taiwan; wsm4@ulive.pccu.edu.tw; 4 Department of Biomedical Engineering, University of Virginia, Charlottesville, VA 22903, USA; yyc0721@gmail.com; 5 Department of Research, Taipei Tzu Chi Hospital, Buddhist Tzu Chi Medical Foundation, New Taipei City 23142, Taiwan; 6 The Division of Cardiology, Department of Internal Medicine and Cardiovascular Center, Taipei Tzu Chi Hospital, Buddhist Tzu Chi Medical Foundation, New Taipei City 23142, Taiwan

**Keywords:** *CDH13*, adiponectin, HDL-C, GWAS, suppression effect

## Abstract

*CDH13* encodes T-cadherin, which is expressed in the vasculature and cardiac myocytes and is the receptor for hexameric and high-molecular-weight adiponectin. The *CDH13* region is the most pivotal locus associated with adiponectin level. Mediation analysis is a method to explore the effect of a third variable, it is assumed that the magnitude of the relationship between the independent and dependent variables will be reduced by statistical adjustment for a third variable. In addition, mediation can further occur in the case when the mediator acts as a pathway-suppressor variable that means a suppression effect may be suggested if the statistical removal of a mediation effect could increase the magnitude of the relationship between the independent and dependent variables. Here, we aimed to explore the suppression effect in a genome-wide association study, and investigate possible mechanisms that may link adiponectin to *CDH13* variants and high-density lipoprotein cholesterol (HDL-C). Genome-wide association data for adiponectin and HDL-C were accessible for 2349 Taiwan-biobank participants. The mediation analysis was conducted with the *CDH13* lead single nucleotide polymorphism (SNP) rs4783244. The cloned constructs of *CDH13* haplotypes (GG and TT) identified from the rs4783244 G/T and rs12051272 G/T SNPs were transiently expressed in HEK293T cells and investigated using the luciferase reporter assay. Genome-wide association analysis showed that HDL-C is significantly associated with variants in *CDH13* after adjusting for the adiponectin level. The lead SNP rs4783244 was significantly associated with lower adiponectin levels and exhibited a suppression effect on HDL-C when adiponectin was included as a third factor in the mediation analysis. Luciferase reporter assay results further demonstrated that the GG haplotype increased enhancer activity, whereas the haplotype TT significantly reduced the activity of this enhancer. We present the first evidence of the suppressive role of adiponectin in the genome-wide association between *CDH13* and HDL-C. *CDH13* may increase the HDL-C levels, and its expression is suppressed by adiponectin.

## 1. Introduction

Adiponectin is one of the most plentiful gene products in adipose tissue. It acts a crucial part in the metabolic arrangement of obesity, insulin sensitivity, and atherosclerosis [1]. Many studies have shown that adiponectin has diverse metabolic functions, such as anti-diabetic, anti-inflammatory, and anti-atherosclerotic activities [2]. It is believed that adiponectin levels are regulated by genetic factors, as demonstrated by twin studies [3], family studies [4], and genome-wide linkage studies [5], which show a moderate to high estimate of heritability (30–70%) [6]. Furthermore, a recent report focusing on family studies found that there is a common heritability between adiponectin levels and metabolic syndrome [7]. Several genome-wide association studies, in which meta-analysis was performed, revealed various candidate gene loci, which may affect adiponectin levels [8,9,10]. The *CDH13* gene, which is located on chromosome 16q23.3, spans 1.2 Mb, contains 14 exons and encodes T-cadherin. A meta-analysis showed that the *CDH13* gene region is the most crucial locus related to adiponectin levels [11]. A *CDH13* intron 1 polymorphism was only associated with the metabolic phenotype reported by two recent studies on East Asian populations after adjusting for the level of high-molecular-weight (HMW) adiponectin [10,12]. Genome-wide association studies (GWAS) in various ethnic groups have found an association between a *CDH13* single nucleotide polymorphism (SNP) and adiponectin and lipid levels [9,10,12,13]. Recent research in human genetics has tended to focus on genes that affect HDL metabolism, and to assess the probable associations of those genes with the risk of coronary heart disease and HDL-C levels. In spite of the fact that physical and environmental factors may alter HDL function and HDL-C levels, it is considered that 40–60% of variations in HDL-C levels can be illustrated by genetic variation [14]. Many research reports using GWAS methods have demonstrated that common mutation association studies can successfully detect common polymorphisms in some genes known to regulate HDL that are related to this variable trait [15]. Nevertheless, it is still very rare that the known sites found by GWAS can explain the mutation sites related to HDL-C levels suggesting that there are likely to be other pathways and target genes influencing HDL-C levels [16,17,18]. Mediation analysis is a technique that is more commonly used in psychology and social sciences [19,20]. It is a model to explore the effect of a third variable, and its ability to influence the relationship of independent variables with dependent variables [21]. The so-called ‘mediation effect’ means that the independent variable has an effect on the mediator, and consequently affects the dependent variable [22]. In mediation analysis, when the direct and indirect effects of the independent variables have opposing actions, a suppression effect occurs [23]. Our previous study of candidate genes in the general population of Taiwan, which included mediation analysis, provided the first evidence of suppression effects in this context [24,25]. The main purpose of this study was to use GWAS to verify the suppression effect identified by the mediation analysis, and to find the lead SNPs in the *CDH13* gene and estimate their impact on HDL-C levels. The second purpose was to estimate the functional activity of the *CDH13* haplotype, to provide additional information on the existence of genetic factors that contribute to the cardiovascular risk associated with the blood lipid profile of Taiwanese individuals in the general population.

## 2. Materials and Methods

### 2.1. Subjects 

The subjects of this study consisted of 2349 subjects from the Taiwan Biobank (TWB), their related information were collected from recruitment centers throughout Taiwan from 2008 to 2015 [26]. Written informed consent was collected from all subjects before enrollment. The recruited participants had no history of coronary artery disease, stroke, cancer, or systemic disease, and a total of 150 subjects were removed from the study according to the following criteria: retraction of informed consent after participation (2 subjects), fasting < 6 hours (38 subjects), and quality control (QC) for the GWAS (110 subjects). The study was approved by The Institutional Review Board of Taipei Tzu Chi Hospital (approval number: 08-XD-011), Buddhist Tzu Chi Medical Foundation. 

### 2.2. GWAS Population

The TWB genotype array for high-throughput Affymetrix Axiom genotyping platform was designed by the Taiwan Biobank study group. SNPs on the Axiom Genome-Wide CHB 1 Array plate (Affymetrix, Inc., Santa Clara, CA, USA) with 5% minor allele frequencies (MAFs) in a set of 2349 samples from the Taiwanese Han Chinese population. With the support of the National Center of Genome Medicine of the Academia Sinica, the genomic DNA was genotyped using the Axiom TM-TWB genome-wide array comprising 646862 SNPs. A call rate of ≥ 97% was noted in all samples for analysis. The QC for SNPs was established as follows: minor allele frequency < 0.05, SNP call rate < 3% and violation of Hardy–Weinberg equilibrium (*p* value < 10^−6^) were excluded from following analyses. A total of 2199 subjects and 614823 SNPs were recruited for the GWAS analysis after QC.

### 2.3. Genomic DNA Extraction and Genotyping

DNA was isolated from blood samples of the TWB participants, using a QIAamp DNA blood kit (Qiagen, Valencia, CA, USA) following the guidance of the manufacturer. SNP genotyping was carried out adopting custom TWB chips, and performed on the Axiom Genome-Wide Array Plate System (Affymetrix, Santa Clara, CA, USA).

### 2.4. Laboratory Examinations and Assays

The clinical phenotypes included body mass index (BMI), body weight, body height, waist-to-hip ratio, waist circumference, and systolic and diastolic blood pressure were selected for analysis. BMI was calculated as body weight (kg)/height (m^2^). Biochemistry data obtained for analysis included fasting plasma glucose, as well as lipid profiles, including TG levels, high-density lipoprotein (HDL), low-density lipoprotein cholesterol (LDL-C), and total cholesterol. The commercial kits for adiponectin (R&D Systems, Minneapolis, MN, USA) was adopted to measure serum adiponectin levels. 

### 2.5. Mediation Analysis

A mediation model was assumed (Figure S1) to investigate the mediation effects of adiponectin levels on the association between the *CDH13* lead SNP and HDL-C, and the suppression effects were confirmed by investigating the following four criteria [19]: Criterion one demonstrates that the association between the independent variable and mediator must be significant (α). Criterion two demonstrates that, after adjusting for the independent variable, the association between the mediator and dependent variable must be significant (β). The calculated product of the two regression coefficients from criterion one and criterion two (αβ) was considered as the mediation (indirect) effect, which expressed the intermediate pathways from the independent variable to the mediator, and in turn to the dependent variable. The regression coefficient connecting the independent variable to the dependent variable when adjusting for the mediator was computed as the direct effect (γ'). Criterion three demonstrates that the total effect (αβ+γ') must be significant, which was expressed as the association between the independent variable and dependent variable. Criterion four demonstrates that the mediation effect which was determined by using the Sobel test and must be significant [21,27,28]. In addition, a status in which the direct effect was larger than the total effect could be exhibited as a suppression effect [29]. Under this status, direct and indirect effects frequently have comparable significances and opposing signs, which may totally or partially abolish each other and lead to a zero or nonzero but insignificant total effect [30].

### 2.6. Construct

A genomic DNA fragment of the *CDH13* haplotype, which enclosed SNPs rs4783244 and rs12051272, was amplified by polymerase chain reaction (PCR) as presented in our former study [25]. The upstream and downstream primers used were 5′-GTACCAGGGGAGATTCAAGTGC-3′ and 5′-CTCTCCACTGACAAGTTGCCAC-3′, respectively. By following the manufacturer’s instruction, PCR products were subcloned into the pEASY-T1 vector (Invitrogen, Carlsbad, CA, USA). Plasmid DNA was subsequently isolated from recombinant colonies and sequenced to ensure accuracy. The *CDH13* haplotype inserts, which contained two haplotypes that identified from rs4783244 G/T and rs12051272 G/T (H1: GG and H2: TT), were subsequently extracted by XhoI and HindIII digestion, gel-purified, and subcloned into the pGL2.0-promoter(Luc 2) vector (Promaga, Madison, WI, USA), upstream of the firefly luciferase reporter gene. The validity and orientation of the inserts relative to the luciferase gene were confirmed by sequencing.

### 2.7. Transfection and Functional Reporter Assay

The Dulbecco’s modified Eagle’s medium (Sigma, CA, USA) supplemented with 10% fetal calf serum (HyClone Laboratories, Logan, UT, USA) was used for culture the HEK-293T cells in 5% CO_2_ at 37℃. Cells were plated in a 6-well plate and grown to 80–90% confluence. The X-treme GENE HP DNA Transfection Reagent (Roche, Mannheim, Germany) was used for transfection and the *CDH13* haplotype constructs (500 ng) were co-transfected with 50 ng pRL-TK (Promega, Madison, WI, USA), which encoded the Renilla luciferase into HEK-293T cells. Using a dual-luciferase reporter assay system (Promega) according to the manufacturer’s instruction, luminescence was measured 48 h after transfection. All data were analyzed by normalizing firefly luciferase activity with that of the Renilla luciferase for each sample.

### 2.8. Statistical Analysis

Statistical analysis was performed as previously described [24]. GWAS analysis was calculated using PLINK. *P* values less than the threshold of *p* = 5×10^−8^ were considered significant genome-wide. All calculations were examined adopting SPSS version 22 (SPSS, Chicago, IL, USA). *P* values <0.05 using a two-sided test were recognized as significant. List-wise deletion was used to manage missing data. The investigation of deviation from the Hardye Weinberg equilibrium, estimation of linkage disequilibrium between polymorphisms, association between haplotypes and lipid-protein levels were executed adopting the GoldenHelix SVS Win32 7.3.1 software (Golden Helix, Bozeman, MT, USA).

## 3. Results

### 3.1. Subsection

#### 3.1.1. Characteristics of the TWB Participants

Clinical phenotypes and biochemical profiles of the subjects, arranged by sex, are demonstrated in Table 1. The men who were current smokers had a significantly higher percentage (*p* < 0.001). Furthermore, the women had significantly lower waist circumference (*p* < 0.001), waist/hip ratio (*p* < 0.001), BMI (*p* < 0.001), and circulating levels of TG (*p* < 0.001) and LDL-C (*p* = 0.002) than the men. In addition, circulating HDL-C (*p* < 0.001) and adiponectin levels (*p* < 0.001) were higher in women than in men. 

#### 3.1.2. Genome Wide Association Study for HDL-C in the TWB Participants

**Figure 1:**
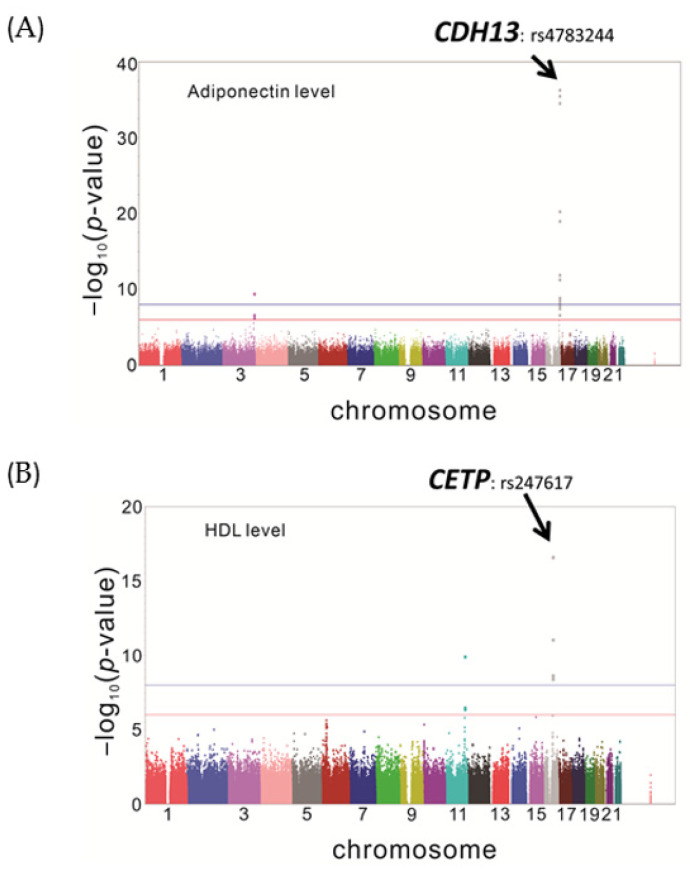

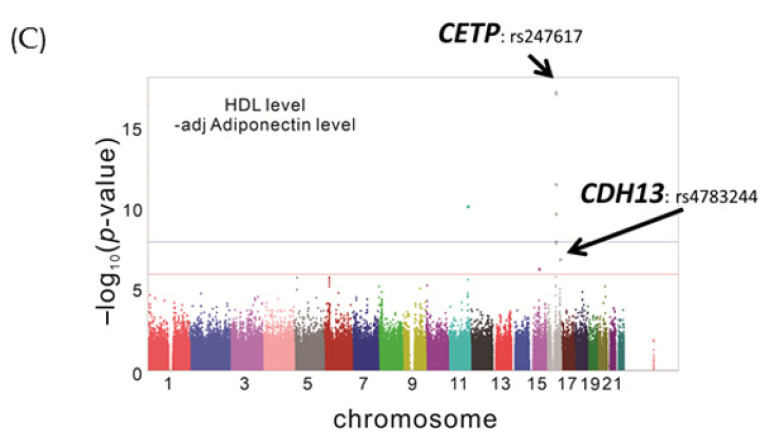
GWAS analysis of Adiponectin and HDL levels. Manhattan plots of genome-wide association study for Adiponectin and HDL levels. (**A**) The data revealed the genome-wide significant gene loci *CDH13* in Taiwanese for Adiponectin levels. *P* value adjusted for age, sex, current smoking status and body mass index. (**B**) The data of the genome-wide significant gene loci *CETP* in Taiwanese for HDL levels. *P* value adjusted for age, sex, current smoking status and body mass index. (**C**) The data revealed the genome-wide gene loci *CDH13* in Taiwanese for HDL levels became significantly after further adjusted for Adiponectin levels.

After examining quality control of the data according to approved procedures, the reports from GWAS analysis revealed that one region of chromosome 16 located in the *CDH13* gene displayed a highly significant association with adiponectin levels (Figure 1A). Furthermore, it was consistent with the results from previous studies, several variants located upstream and in the 5′-UTR of the cholesteryl ester transfer protein *(CETP)* gene on chromosome 16, had a highly significant association with HDL-C levels (Figure 1B). Interestingly, another region of chromosome 16, located in the *CDH13* gene (Figure 1C), was significantly associated with HDL-C levels after adjustment for adiponectin levels. Taking into account adjustment for adiponectin, the most statistically significant SNP was rs4783244 (Table 2), demonstrating the adiponectin strongly suppressed the association between HDL-C and *CDH13* gene variants. 

#### 3.1.3. Mediation Analysis of the Association between HDL-C and rs4783244 in the TWB Population

Four criteria were examined to demonstrate mediation and suppression effects (Table 3). After adjustment for BMI, age, sex, and smoking, the *CDH13* SNP rs4783244 was noted to be significantly related to lower adiponectin levels (*p* = 3.68 × 10^−37^) (criterion 1), which consecutively had significant effects on HDL-C levels (*p* = 1.12 × 10^−93^) (criterion 2). The total effect of rs4783244 on HDL-C levels was −0.001 (*p* = 0.731) (criterion 3). The Sobel test for mediation on the results of HDL-C displayed a z value of −11.16, and a *p* value of < 10^−8^ (criterion 4). Moreover, the direct effect (γ') of rs4783244 on HDL-C levels (0.015) was larger than the total effect (αβ+γ') (−0.001) and showed the opposite response with the mediation effect (αβ) (−0.017), indicating a suppression effect in this context.

#### 3.1.4. Functional Analysis of Two Haplotypes in the *CDH13* Proximal Intron 1 Promoter Region

**Figure 2:**
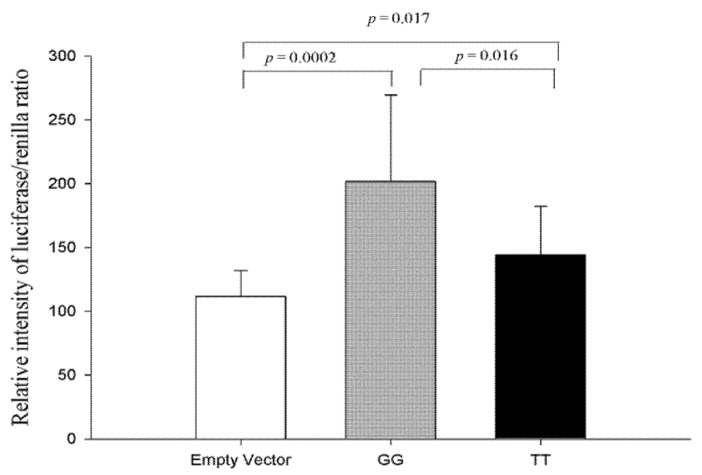
Luciferase reporter assay of pGL2.0-promoter-GG and TT in HEK293T cells. Activity of *CDH13* haplotype H1: GG significantly increased 80% (*p* = 0.0002, 95% confidence interval [−132.19, −47.76]) as compared with empty vector (pGL2.0-promoter). Furthermore, the activity of *CDH13* haplotype H2: TT significantly decreased 28% (*p* = 0.016, 95% confidence interval [11.19, 104.04]) as compared with *CDH13* haplotype H1: GG. Data were normalized to renilla activity and expressed as percentage of empty vector. H1: GG (haplotype of rs4783244 G and rs12051272 G); H2: TT (haplotype of rs4783244 T and rs12051272 T).

To investigate whether the haplotypes of rs4783244 G/T and rs12051272 G/T regulate the enhancer activity of the promoter, we expressed them in a luciferase reporter construct in HEK293T cells and analyzed luciferase activity in the cell lysates. The H1: GG revealed significantly increased enhancer activity (*p =* 0.0002) after transfection when compared with the results of the control (pGL2-promoter vector). Furthermore, the H2: TT significantly reduced enhancer activity compared to the major haplotype H1: GG (*p =* 0.016) (Figure 2). These results demonstrated that the *CDH13* haplotypes are functional, and H2: TT exhibits a crucial role in the regulation of *CDH13* expression.

## 4. Discussion

In this study, we examined the suppressive role of adiponectin in the association between the *CDH13* gene and HDL-C. To verify the suppression effect identified in our prior candidate gene study, we investigate a GWAS in a larger Taiwanese participant. Consistent with our previous study, the *CETP* gene located in chr.16 was noted to be strongly associated with HDL-C [31]. Furthermore, performing a GWAS on adiponectin revealed a strong association with SNPs in *CDH13*. This finding is in line with former studies in Japanese, Singaporean Chinese, and Korean populations [10]. However, several GWAS performed on populations in Western countries have shown that a major effect on plasma adiponectin levels is exerted by *ADIPOQ* [7,32,33]. Studies on genome-wide associations of adiponectin have demonstrated ethnic differences between Asian and Western populations, while the effects of *CDH13* promoter variants also vary between Asian and European populations [9,12,13,34]. The minor allele frequency (MAF) of rs4783244 in the Human HapMap project was revealed to be high similarly in both Caucasians and Han Chinese (0.46 and 0.32), whereas the MAF of rs12051272 was much lower in Caucasians (0.009) than in Han Chinese (0.22). A previous study showed that American men exhibited higher adiponectin levels than the Japanese men in spite of higher levels of obesity [35]. This might be due to the fact that most of the Americans in this study may have carried the adiponectin-increasing allele rs12051272-G, resulting in higher mean plasma adiponectin levels.

**Figure 3:**
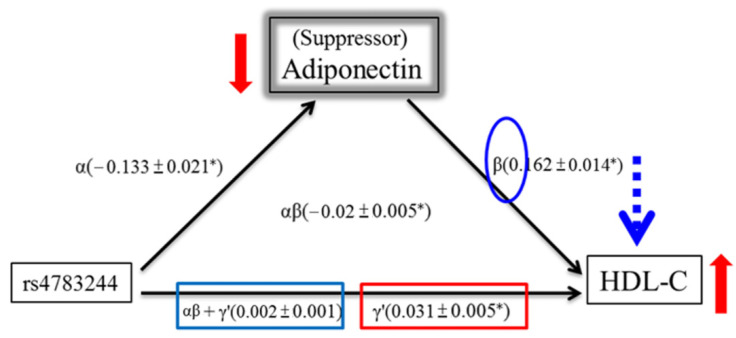
Suppression effect of Adiponectin. A three-variable mediation model: Adiponectin level as a suppressor of association between *CDH13* genotype and HDL-C. Linear regression model was used to assess the path associations of α,β,γ', and αβ+γ'.

We discovered that several SNPs in the 5′-UTR of *CDH13* were significantly associated with HDL-C after adjustment for adiponectin levels. This report is in line with those from a previous study [10]. In the current study, we successfully applied the mediation model and conducted mediation analysis of the *CDH13* lead SNP rs4783244, confirming that adiponectin exerts a suppression effect on the association between *CDH13* and HDL-C levels. As summarized in Figure 3, since the carriers of the minor genotype T express low adiponectin levels (red arrow), we hypothesize that the association between the *CDH13* genotype T and HDL-C is suppressed by adiponectin due to the positive correlation (blue circle) between adiponectin level (red arrow) and HDL-C level (red arrow). This reduces the HDL-C level (blue arrow) and, therefore, masks the significant association (red square) between the *CDH13* genotype T and HDL-C into non-significance (blue square).

The results of the luciferase reporter assay additionally demonstrated that the haplotypes of rs4783244 G/T and rs1205172 G/T were functional, and that the functional enhancer activity of the common haplotype H1: GG can be reduced by the minor haplotype H2: TT, comprising the adiponectin-decreasing alleles, rs4783244T and rs1205172T. The mechanism through which *CDH13* SNPs may affect the expression of the *CDH13* gene had been speculated on in two separate publications. First, Spracklen et al. reported that rs12051272 is located in *CDH13* intron 1, approximately 3 kb from the transcription start site, in an accessible chromatin region with chromatin marks of active enhancers in Hela cells. Furthermore, results from their reporter assays showed that the allele rs12051272-G displayed an average of 1.7-fold increased enhancer activity compared to the rs12051272-T allele [13]. Second, Putku et al. reported that several SNPs around the CpG island in the regulatory region of the *CDH13* gene influence the methylation levels of the gene region. The authors showed that two SNPs (rs8060301 and rs12444338) were related to hypermethylation within the CpG island, and that rs4783244 and rs12051272 were located near rs8060301 and rs12444338 in linkage disequilibrium. Therefore, they are considered to influence the methylation and expression of *CDH13* [35].

The suppression effect of mediation analysis is not often used in genetic association studies. In this study, we used a second, larger sample size of the Taiwanese population to conduct GWAS and successfully replicated our initial finding regarding the suppression effect of adiponectin. A study by Yamauchi et al., proposed that the adiponectin R1/R2 receptors confer almost all the metabolic effects of adiponectin [36]. However, adiponectin can also bind to another receptor (T-cadherin) to initiate the nuclear factor-kB signaling pathway, which exhibits a vital part in inflammation and is an important association between vascular disease and obesity [37]. Indeed, T-cadherin competes with the adiponectin R1/R2 receptors to bind adiponectin, and it also intervenes with the coupling of the two receptors to their downstream intracellular goals [38].

Taking into account the combined findings of the mediation analysis and functional activity assays, we hypothesize that the haplotype TT of rs4783224 T and rs12051272 T at the *CDH13* locus may decrease the expression of the *CDH13* gene and T-cadherin protein, resulting in decreased competition with other adiponectin receptors and increased coupling of adiponectin R1/R2 receptors with their downstream intracellular targets, in addition to the metabolic effects of adiponectin. As formerly suggested [10], increased adiponectin sensitivity and adiponectin R1/R2 expression may be associated with chronic low levels of adiponectin. Higher expression of the adiponectin receptors could counterbalance low adiponectin levels, and lead to a non-significant association between rs4783244 and HDL-C levels in unadjusted analyses. Nonetheless, when circulating adiponectin is controlled for, then greater “adiponectin sensitivity” leads to a significant association between the T allele and more favorable HDL-C levels. This hypothesis is supported by the results of our mediation analysis, which indicates that adiponectin acts as a suppressor of the association between the *CDH13* genotypes/haplotypes and HDL-C levels. Even though the current study presented a probable mechanism for *CDH13* gene regulation, it will still be crucial to set up an animal model in which to verify this suppressive effect in vivo. 

One of the limitations of this research is that we did not check HMW adiponectin, particularly as T-cadherin has been recognized as an adiponectin-binding protein with a preference for HMW adiponectin. Furthermore, the SNPs in *CDH13* are more strongly associated with HMW compared with total adiponectin. Therefore, further research is required to examine the correlation between the *CDH13* SNPs and HDL-C with respect to HMW adiponectin levels. Another limitation was that the sample size used for the GWAS analysis in this study was medium. Future studies will need to employ larger-scale GWAS to obtain stronger *p* values for the suppression effect. 

## 5. Conclusions

We demonstrate the first evidence of the suppressed role of adiponectin in the association between *CDH13* and HDL-C. We also present that the *CDH13* haplotype is functional and suggest that the reduced *CDH13* activity found in carriers of H2: TT is likely due to reduced transcription.

## Figures and Tables

**Table 1:** Clinical phenotypes and biochemical profiles of the TWB participants.

	Total	Men	Women	*p* value
Number	2199	986	1213	
Age (years)	48.1 ± 10.8	48.6 ± 11.1	48.2 ± 10.7	0.444
BMI (kg/m^2^)	24.2 ± 3.5	25.1 ± 3.4	23.4 ± 3.5	<0.001
Waist circumference (cm)	84.8 ± 9.4	87.4 ± 9.2	80.4 ± 9.2	<0.001
Waist-to-hip ratio	0.9 ± 0.2	0.90 ± 0.2	0.84 ± 0.1	<0.001
Current smokers (%)	396 (18.0%)	329 (33.4%)	67 (5.5%)	<0.001
Total cholesterol (mg/dL)	195.1 ± 35.9	194.6 ± 35.4	195.6 ± 36.5	0.500
LDL-C (mg/dL)	122.5 ± 32.9	124.8 ± 32.	120.5 ± 32.7	0.002
HDL-C (mg/dL)	55.1 ± 13.4	49.7 ± 11.4	59.4 ± 13.3	<0.001
TG (mg/dL)	117.9 ± 91.9	138.9 ± 105.8	100.8 ± 74.7	<0.001
Adiponectin (mg/L)	3.5 ± 1.9	2.88 ± 1.45	4.01 ± 1.97	<0.001

BMI, body mass index; LDL-C, low-density lipoprotein cholesterol; TG, triglycerides; HDL-C, high-density lipoprotein cholesterol. Continuous variables are expressed as means ± SD. Before statistical testing, HDL-C and TG values were logarithmically transformed to adhere the assumption of normal distributions; nonetheless, the untransformed data are presented here.

**Table 2:** Association of *CDH13* SNPs with HDL level before and after adjusted for adiponectin.

CHR	Gene	SNP	Chr. Location	Minor Allele	*p*	*p* *
16	CDH13	rs4783244	82,662,268	T	0.8439	1.05 × 10^−7^
16	CDH13	rs28597883	82,647,566	C	0.4147	7.66 × 10^−6^
16	CDH13	rs3865188	82,650,717	T	0.3587	7.84 × 10^−6^
16	CDH13	rs12444222	82,672,920	G	0.1005	1.40 × 10^−5^
16	CDH13	rs4783248	82,674,055	G	0.11	2.29 × 10^−5^
16	CDH13	rs12446731	82,676,142	A	0.1179	3.28 × 10^−5^
16	CDH13	rs12925602	82,655,901	A	0.1391	5.01 × 10^−5^
16	CDH13	rs9652670	82,671,477	G	0.1597	7.10 × 10^−5^
16	CDH13	rs3865186	82,646,972	A	0.3461	7.90 × 10^−5^
16	CDH13	rs9940180	82,653,444	T	0.9354	8.00 × 10^−5^

*p*: adjusted for age, sex, BMI, and smoking; *p* *: adjusted for age, sex, BMI, smoking, and Adiponectin.

**Table 3:** Mediation analysis of adiponectin levels on the association between *CDH13* SNP rs4783244 and HDL-C levels.

		rs4783244
Criterion 1	α	
	regression coefficient	−0.079
	Standard error	0.006
	*p* value	3.68 × 10^−37^
Criterion 2	β	
	regression coefficient	0.21
	Standard error	0.01
	^#^*p* value	1.12 × 10^−93^
	γ′	
	regression coefficient	0.015
	Standard error	0.003
	* *p* value	7.17 × 10^−8^
Criterion 3	αβ + γ′	
	regression coefficient	−0.001
	Standard error	0.003
	* *p* value	0.731
Criterion 4	αβ	
	regression coefficient	−0.017
	Standard error	0.001
	*p* value (Sobel test)	<10^−8^

α: unstandardized coefficient for the association between *CDH13* SNP rs4783244 and adiponectin levels. β: unstandardized coefficient for the association between adiponectin and HDL-C levels (adjusted for rs4783244). Direct effect = γ′, Total effect = αβ+γ′, Mediation (indirect) effect = αβ. *p*: adjusted for BMI, age, sex, current smoke; *: *p* value of association between rs4783244 and HDL-C after adjustment for age, sex, BMI, current smoke, and adiponectin; ^#^: *p* value of association between adiponectin and HDL-C after adjustment for BMI, age, sex, current smoke, and rs4783244.

## Supplementary Materials

The following is available online at https://www.mdpi.com/article/10.3390/genes12101582/s1, Figure S1: A conceptual model for mediation analysis.
