# Supplementary material for: Genome-Wide Association Study on Adiponectin-Mediated Suppression of HDL-C Levels in Taiwanese Individuals Identifies Functional Haplotypes in CDH13
Source: Genes (Basel). 2021 Oct 7;12(10):1582. doi: 10.3390/genes12101582 (PMC8535967; doi:10.3390/genes12101582)
Supplement: Supplementary file 1 [file genes-12-01582-s001.zip › genes-1386675-supplementary.pdf]

Supplementary Figure S1. A conceptual model for mediation analysis

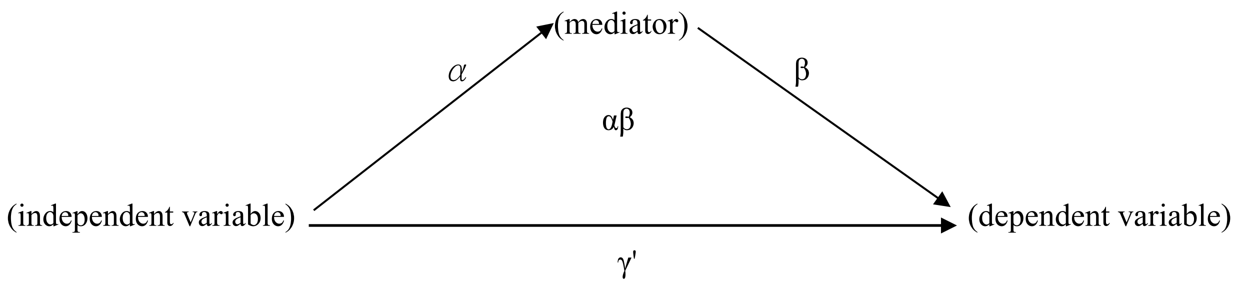

α : unstandardized coefficient for the association between independent variable and mediator.

β : unstandardized coefficient for the association between mediator and dependent variable (when adjusting for independent variable.)

Mediation (indirect) effect = $\alpha\beta$; Direct effect = γ'; Total effect = $\alpha\beta + \gamma'$
